# Melphalan Culprit or Confounder in Acute Encephalopathy during Autologous Hematopoietic Stem Cell Transplantation?

**DOI:** 10.1155/2012/942795

**Published:** 2012-05-07

**Authors:** Diógenes Alayón-Laguer, Melissa Alsina, Jose L. Ochoa-Bayona, Ernesto Ayala

**Affiliations:** ^1^Hematology-Oncology Program San Juan City Hospital and VA Caribbean Healthcare System, 10 Calle Casia, San Juan, PR 00921, USA; ^2^Blood an Marrow Transplantation Program, Moffitt Cancer Center, Tampa, FL 33612, USA

## Abstract

We report a case of a female patient with Durie-Salmon stage 3A/ISS stage I IgG kappa multiple myeloma (MM) who developed encephalopathy after high-dose melphalan and hematopoietic stem cell transplant (HSCT). The most common etiologies for encephalopathy such as infection, narcotic medications, metabolic-electrolyte disturbance, stroke, and central nervous system (CNS) hemorrhages were ruled out. The patient recovered from the altered mental status spontaneously. The possibilities of melphalan-induced encephalopathy versus critical-state delirium versus hypercytokinemia induce encephalopathy were contemplated.

## 1. Introduction

Neurological complications associated to hematopoietic stem cell transplant (HSCT) encompass distinctive entities which can involve the central nervous system (CNS) and the peripheral nervous system (PNS). Among the most commonly described in literature are CNS infections (viral, fungal, and bacterial), metabolic encephalopathies (electrolytes disturbance, febrile and toxic), vascular adverse events (ischemic or hemorrhagic), tumor relapse, and polyradiculoneuropathies [1–3]. Various syndromes are also seen in close relation to the HSCT setting, as are posterior reversible encephalopathy syndrome (PRES) and Wernicke's encephalopathy (WE) [4, 5]. 

 Identifying precipitating factors is crucial on every patient with altered mental status. Melphalan has been linked to some fatal cases of encephalopathy in the setting of chronic kidney disease (CKD) on hemodialysis, during conditioning regimen for HSCT [6]. Higher dose of melphalan administered to hemodialysis patients has been linked to more adverse neurological events than the renal-adjusted dose [6, 7]. 

We present a case of unspecified encephalopathy after a high-dose melphalan conditioning regimen followed by autologous hematopoietic stem cell transplant (Auto-HSCT). Possible etiologies, evaluation and therapeutic modalities would be discussed.

## 2. Case History

60-year-old obese African American female with past medical history of diabetes mellitus type II, hypertension, gout, vertigo, hyperlipidemia, peripheral neuropathy, and CKD stage II (glomerular filtration rate of 88 mL/min) after MM diagnosis presented with a left groin mass. Biopsy was consistent with light-chain- (LC-) restricted plasmacytoma with associated amyloid deposition. Further workup showed an IgG kappa serum M-spike of 3.2 g/dL and multiple lytic lesions in bone survey without hypercalcemia or anemia. B2 microglobulin and albumin were 2.5 mg/L and 3.9 g/dL, respectively. Bone marrow aspiration and biopsy showed 25% plasma cells with 13q deletion and 1+ by both cytogenetics and fluorescence in situ hybridization (FISH). The patient was therefore diagnosed as having high-risk Durie-Salmon stage IIIA with an ISS stage I multiple myeloma. She received induction therapy with 4 cycles of lenalidomide, bortezomib, and dexamethasone, with a very good partial response. The patient was then evaluated for autologous stem cell transplant, and pretransplant workup was remarkable for CKD stage II, albumin at 3.2 g/dL, and a Karnofsky performance status of 80%. Stem cells were mobilized with Neupogen, and a total of 6.17 mill/kg CD 34 cells were collected. The patient was then admitted to the hospital and received high-dose melphalan (200 mg/m²), followed by stem cell infusion.

White blood cell (WBC) nadir occurred on day +6 with engraftment documented by day +13. The treatment-related toxicities included grade 3 mucositis and grade 2 diarrhea which started by day +7 after the Auto-HSCT. Supportive therapy was provided with pain management, aggressive electrolyte repletion, and antidiarrheal medications. These toxicities improved by day +11. On day +12 the patient developed slow mentation, which progressed rapidly over the next few days resulting in poor contact with reality, verbalizing incomprehensible sounds, inability to follow commands, and responsiveness only to painful stimuli. The Glasgow coma scale at that time was 10/15, (4 for spontaneous eye opening, 2 for incomprehensible sounds at verbal, and 4 while withdraws of pain). A brain MRI with and without contrast was performed and was within normal limits. Characteristic imaging findings of PRES were not identified on MRI [4]. Lumbar puncture for cerebral spinal fluid (CSF) evaluation was performed and herpes simplex virus, human herpes virus 6, toxoplasmosis, *Cryptococcus*, West Nile virus, JC virus, St. Louis encephalitis, and Eastern equine encephalitis were ruled out. Cytomegalovirus polymerase chain reaction (PCR) was positive for less than 250 copies, the same as in serum. CSF cytology was negative for malignancy. Electroencephalogram (EEG) was consistent with nonspecific encephalopathy based on generalized slowing and disorganization without focal features. Over the next 8 days the patient showed no improvement and was restless, anxious, and tachypneic. No evidence of cranial nerve deficit or lung parenchymal disease was observed. All medications with potential psychotropic effect were discontinued at the onset of the encephalopathic symptoms. No metabolic abnormalities were identified, and the patient was started on parenteral nutrition. Thiamine 100 mg IV daily was provided upon consideration of Wernicke's encephalopathy. The possibility of ammonia-induced encephalopathy even with normal liver function tests (LFTs) was contemplated, but levels returned <200 *μ*M/L. Several factors of distress were evident which included a sudden increase at WBC even while off Neupogen, an isolated episode of nonneutropenic fever and evidence of nonoliguric acute kidney injury (creatinine increased from baseline of 1 mg/dL to a maximum of 1.7 mg/dL for a glomerular filtration rate of 52 mL/min.). Systemic evaluation for infectious process (viral, fungal, and bacterial) was also performed repeatedly without an isolated organism identified. Empirical broad-spectrum antimicrobial coverage against opportunistic and most commonly related infectious process in HSCT patients was administered. The therapy provided consisted of vancomycin, cefepime, micafungin, and metronidazole, in descalating fashion upon correlation of the negative panculture evaluation.

On day +20 the patient suddenly recovered from the altered mental status without neurologic sequelae. Supportive therapy was provided to regain patient performance and strength. Upon interrogation she denied remembering any of the events. Her last recollection of event was back to the day before the mental status changes started. The patient was discharged home by day +26 of the Auto-HSCT with improvement of performance status and no metabolic or neurologic disturbances.

## 3. Discussion

Neurological complications are common after HSCT. These vary according to the underlying disease, type of transplant, and conditioning regimen [1], usually associated with poor survival with the most common etiologies identified in Auto-HSCT being tumor relapse, and in Allo-HSCT CNS infection [1].

 Three main etiologies were considered as the responsible for the idiopathic encephalopathy in our patient after careful evaluation of the case: delirium, melphalan-induced toxicity, and hypercytokinemia. Delirium is a well-described cause of altered mentation after HSCT. It was considered after all the possible organic etiologies of the altered mental status were ruled out. A study of the Fred Hutchinson Cancer Research Center evaluated the risk factors pre- and post-HSCT to be considered as predictive markers for high-risk patients [8]. The study revealed the factors most commonly identified in patients who presented with delirium after transplant with close attention provided to cognitive, liver, and renal impairment [8]. As post-transplant risk factors, inadequately control pain led to the delirium severity while opioid medication was associated to delirium onset [8]. Other alternatives to overuse opioid medications are recommended to prevent delirium onset. Our patient was thought to have delirium related to opioid and psychotropic medications after transplant. Supportive therapy was provided, and all medications were withdrawn immediately with the onset of symptoms. The expected recovery after 72 hours of all medications discontinue did not occur leading us to consider some other etiology as the cause of the encephalopathy.

The infusion of high-dose melphalan led to consider the drug as the culprit of the acute encephalopathy. Mlephalan toxicity profile includes CNS depression, decreased consciousness, nausea, vomiting, seizures, and muscle paralysis at doses of 290 mg/m^2^ [9]. Melphalan exerts its effect bound to plasma proteins; 40 to 60% is bound to albumin leading patients with hypoalbuminemia clear the medication faster [10]. In literature one report by Kergueris et al. showed that patients with normal renal function could eliminate melphalan less efficiently due to large interindividual variation among patients [11]. No dose adjustment is recommended at studies of drug pharmacokinetics in patients with early-stage renal insufficiency [10, 11]. In our patient melphalan dose was not adjusted for renal function. With negative workup for encephalopathy and taking in consideration the Kergueris report of unspecified interindividual variation led us to consider melphalan as the culprit.

 Hypercytokinemia-induced encephalopathy was described in a case report of a patient after high-dose melphalan [12]. This encephalopathy was mainly related to tumor necrosis factor alpha (TNF-*α*) effect at brain through stimulation of glial cells and through the tumor necrosis factor receptor 1 (TNFR1) [12, 13]. Endotoxemia through the TNF-*α* interaction with the TNFR1 leads to inflammation in brain, with alteration in blood-brain barrier, upregulation of aquaporin 4 (AQP4-associated edema), neutrophil infiltration, astrocytosis, as well as apoptotic cellular death through nitric oxide mechanism [13]. Blood purification techniques have been studied as anticytokine therapy. The most studied techniques are hemofiltration, continuous hemodialysis, and continuous hemodiafiltration to modulate the inflammatory response in septic patients [14]. Some other nonrenal indications for continuous renal replacement therapy with the intention to remove inflammatory mediators were described in literature [15]. The combined modality of plasma exchange with continuous hemodiafiltration in the setting of critically ill patients was also evaluated with documented benefit in patients with hypercytokinemia [16]. The hemodiafiltration procedure was reevaluated through the use of a poly (methyl methacrylate) membrane hemofilter in 2010 by Nakamura et al. with evidence of adequate removal of proinflammatory cytokines from blood stream in critical care patients [17]. Focosi described a case of plasma exchange response in hypercytokinemia after melphalan conditioning regimen and HSCT [12]. The plasma exchange would remove pathogenic endotoxins, protein binding agents, and immune complexes while replenish coagulation factors or albumin [16]. It was never considered to order cytokine levels prior admission and during acute phase of the event, the reason why this diagnosis was not ruled out in our patient.

The etiology of the encephalopathy was not identified. Multiple factors need to be evaluated in the setting of HSCT when encephalopathy presents. The hypercytokinemia hypothesis needs to be evaluated through prospective clinical trials of patients without and with renal insufficiency after myeloablative conditioning regimens for HSCT. The evidence at two case reports of encephalopathy after melphalan conditioning regimen for an Auto-HSCT [6, 12] in patients with CKD on hemodialysis led to consider the decreased excretion through kidneys as a possible etiology to toxic adverse events.

This case collects the available data of patients who suffer an encephalopathy in the setting of Auto-HSCT with melphalan conditioning regimen. Even when we could not find the etiology of the encephalopathy in our case, we hope this literature review helps others with similar cases for early evaluation, identification of the etiology, and treatment.
